# Effect of formaldehyde exposure on bacterial communities in simulating indoor environments

**DOI:** 10.1038/s41598-021-00197-7

**Published:** 2021-10-18

**Authors:** Jianguo Guo, Yi Xiong, Taisheng Kang, Hua Zhu, Qiwen Yang, Chuan Qin

**Affiliations:** 1 NHC Key Laboratory of Human Disease Comparative Medicine, Institute of Laboratory Animal Sciences, CAMS&PUMC, Pan Jia Yuan Nan Li No. 5, Chao Yang District, Beijing, 100021 China; 2 Key Laboratory of Human Diseases Animal Model, State Administration of Traditional Chinese Medicine, Beijing, 100021 China; 3 Department of Food Science and Engineering, School of Chemistry and Chemical Engineering, Harbin Institute of Technology, Harbin, 150001 China; 4 Department of Clinical Laboratory, Peking Union Medical College Hospital, Peking Union Medical College, Chinese Academy of Medical Sciences, Beijing, 100730 China

**Keywords:** Air microbiology, Occupational health, Environmental impact

## Abstract

Indoor formaldehyde (CH_2_O) exceeding the recommended level is a severe threat to human health. Few studies have investigated its effect on indoor surface bacterial communities, affecting habitants' health. This study used 20-L glass containers to mimic the indoor environment with bacterial inputs from human oral respiration. The behavior of bacterial communities responding to CH_2_O varied among the different CH_2_O levels. The bacterial community structure significantly changed over time in the 0.054 mg·m^−3^ CH_2_O group, which varied from the 0.1 mg·m^−3^ and 0.25 mg·m^−3^ CH_2_O groups. The Chao1 and Shannon index significantly increased in the 0.054 mg·m^−3^ CH_2_O group at 6 week, while they remained unchanged in the 0.25 mg·m^−3^ CH_2_O group. At 12 week, the Chao1 significantly increased in the 0.25 mg·m^−3^ CH_2_O group, while it remained unchanged in the 0.054 mg·m^−3^ CH_2_O group. Only a few Operational Taxonomic Units (OTUs) significantly correlated with the CH_2_O concentration. CH_2_O-induced OTUs mainly belong to the Proteobacteria and Firmicutes. Furthermore, bacterial communities formed at 6 or 12 weeks differed significantly among different CH_2_O levels. Functional analysis of bacterial communities showed that inferred genes related to chemical degradation and diseases were the highest in the 0.25 mg·m^−3^ CH_2_O group at 12 weeks. The development of nematodes fed with bacteria collected at 12 weeks was applied to evaluate the bacterial community's hazards. This showed significantly impaired growth in the 0.1 mg·m^−3^ and 0.25 mg·m^−3^ CH_2_O groups. These findings confirmed that CH_2_O concentration and exposure time could affect the indoor bacterial community and formed bacterial communities with a possibly more significant hazard to human health after long-term exposure to high CH_2_O levels.

## Introduction

People spend most of their time indoors, which is up to 90% in industrialized countries^1,2^. The indoor environment is closely related to human health. There are different air pollutants, including particulate matter^3^, Volatile Organic Compounds (VOCs)^4,5^, microbial contaminants^6,7^, which could affect the morbidity of pneumonia^8^, asthma^9,10^, Chronic Obstructive Pulmonary Disease (COPD)^3^. The interaction among different kinds of air pollutants could not be overlooked, especially between VOCs and microbes. Microbes could produce various VOCs^11^, affecting microbiota serving as carbon sources convinced in plant–microbe interactions research^12,13^. However, indoor VOCs mainly come from types of building materials and furnishings. Few studies have focused on the effect of indoor VOCs on indoor bacterial communities.

Formaldehyde (CH_2_O) is a harmful VOC pollutant in the indoor environment, emitted from various materials, such as CH_2_O-related adhesives^14^, paints, and insulation materials^15^, classified as carcinogenic for humans^16^. A summary of CH_2_O levels for > 2000 residential buildings across 22 cities in China showed that at many locations, the CH_2_O level exceeded the average 30-min level of 100 μg/m^3^ recommended by the World Health Organization^17^. Monitors of CH_2_O positioned at public places also showed that many sampling sites exceeded this level^18^. In our daily life, we perform ventilation to refresh indoor air. However, the microbes attached to the indoor surface could not be easily removed, exposed to high (closed for hours)-low (after ventilation) concentration cycles. These microbes may enter the indoor air by human activity and be inhaled by them. Evidence has shown bacterial community type could affect the morbidity of respiratory diseases^19^. Thus, whether CH_2_O could impact the indoor microbial community and whether CH_2_O could change the microbial community's health risk are major issues. This study aimed to explore the bacterial community succession and evaluate bacterial communities' health risks exposed to high and long gaseous CH_2_O concentration within the scope of monitored concentration in Chinese dwellings^17^.

**Figure 1:**
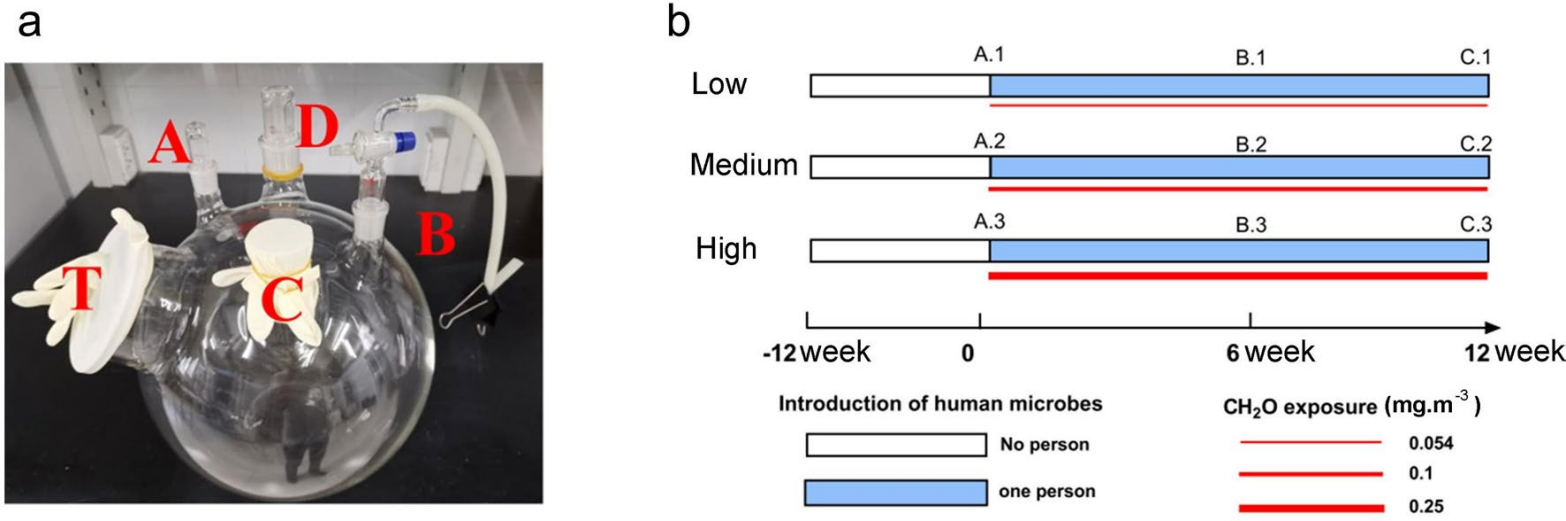
Containers used for simulation of the indoor environment (**a**). Sterile clean 90-mm dishes (without agar) were placed inside each container through the hole T. Hole A for CH_2_O injection, hole B for human input, hole C for simulating nature ventilation, and hole D for sampling. Schematic of experimental operation and grouping (**b**).

In this study, we mimicked the indoor environment using 20-L glass containers and human microbial inputs by occupants via oral respiration, which brings in a much higher number of taxa than via nasal respiration (700 vs*.* 100 taxa)^20,21^ (Fig. 1a). These containers were exposed to three CH_2_O levels and samples were taken at different times (Fig. 1b). Then, the variation of bacterial communities with time among different CH_2_O levels and the bacterial community structure exposed to different CH_2_O levels at 6 or 12 weeks were compared. Finally, the exposed bacterial community's health risk using functional prediction analysis and nematode development experiment was evaluated. This study is valuable for studying the interaction between various VOCs/VOCs complex and indoor bacterial communities.

## Results

### Variation of bacterial communities among different CH_2_O levels

**Figure 2:**
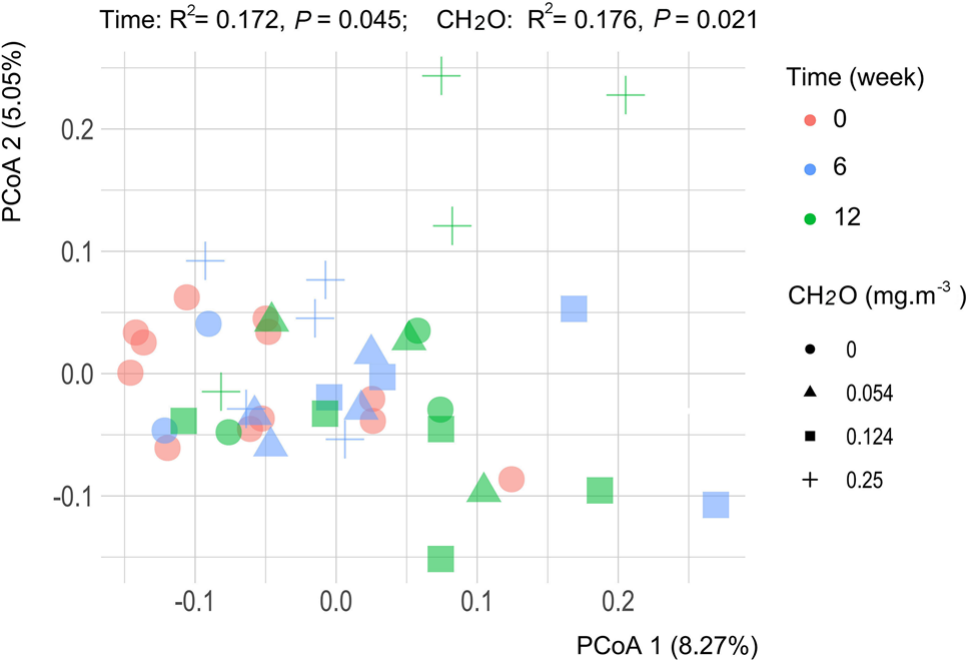
Principal coordinates analysis of bacterial communities based on the Bray–Curtis distance. Permutational multivariate analysis of variance were conducted according to experiment time and CH_2_O levels, respectively. The values of R^2^ and *P* were shown in the figure.

Comparing the bacterial communities in the containers before CH_2_O exposure and input of human oral bacteria showed no significant difference; thus, confirming the bacterial community consistency among these groups (Supplementary Table S1). Principal coordinates analysis based on the Bray–Curtis distance of OTU matrix showed experiment time (PERMANOVA, R^2^ = 0.172, *P* = 0.045) and CH_2_O level (PERMANOVA, R^2^ = 0.176, *P* = 0.021) both significantly affected the bacterial community structure (Fig. 2).

**Figure 3:**
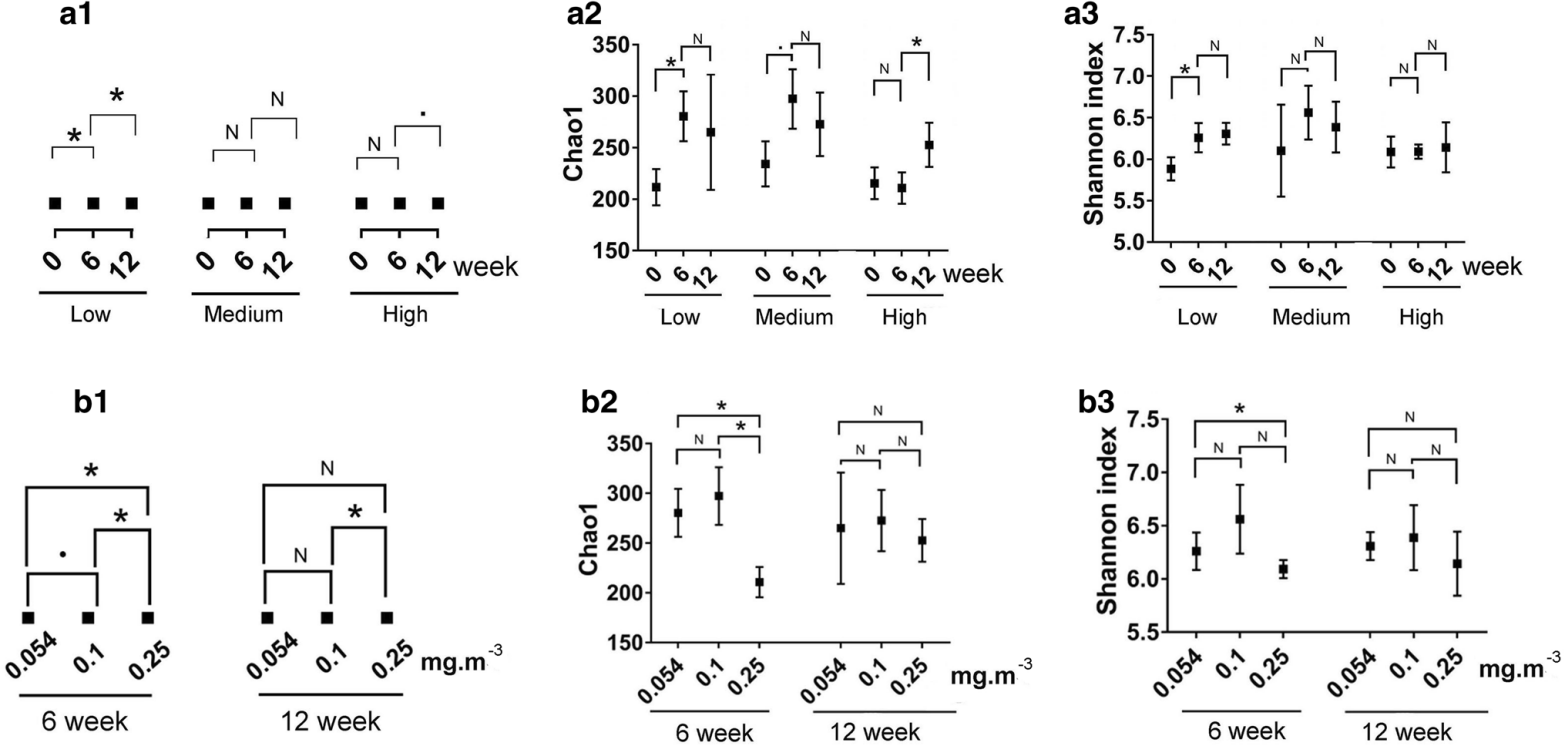
Variations of bacterial communities exposed to different CH_2_O levels. Permutational multivariate analysis of variance of the bacterial community according to exposure time based on Bray–Curtis distances (**a1**), trends of Chao1 (**a2**), and Shannon index (**a3**); Permutational multivariate analysis of variance of the bacterial community according to CH_2_O levels at six and 12 weeks based on Bray–Curtis distances (**b1**), comparation of Chao1 (**b2**) and Shannon index (**b3**) among different CH_2_O levels at six and 12 weeks. Mann–Whitney* U* test was performed. *N* no difference, ^·^*P* < 0.1, *P < 0.05, **P < 0.01. Mean ± std were shown in (**a2**), (**a3**), (**b2**), (**b3**).

We analyzed the temporal variation of bacterial communities among different exposed CH_2_O levels. The indoor bacterial community responded differently to CH_2_O exposure and human oral bacteria (Fig. 3a1). The bacterial community of the 0.054 mg·m^−3^ CH_2_O group significantly changed at 6 (PERMANOVA based on Bray–Curtis distance, R^2^ = 0.192, *P* = 0.029) and 12 weeks (PERMANOVA based on Bray–Curtis distance, R^2^ = 0.198, *P* = 0.026), but that of the 0.1 mg·m^−3^ group did not change at these periods and 0.25 mg·m^−3^ CH_2_O group obviously changed at 12 weeks (PERMANOVA based on Bray–Curtis distance, R^2^ = 0.156, *P* = 0.055) (Fig. 3a1 and Supplementary Table S2). The bacterial diversity index also showed different trends in the different CH_2_O levels (Fig. 3a2,a3). The Chao1 value increased after 6 weeks in the 0.054 mg·m^−3^ (Mann–Whitney *U* test, *P* < 0.05) and 0.1 mg·m^−3^ (Mann–Whitney *U* test, *P* < 0.1) CH_2_O groups, yet did not change in the 0.25 mg·m^−3^ CH_2_O group (Fig. 3a2). However, it showed no change in the 0.054 mg·m^−3^ and 0.1 mg·m^−3^ CH_2_O groups but significantly increased in the 0.25 mg·m^−3^ CH_2_O group after 12 weeks (Mann–Whitney *U* test, *P* < 0.05) (Fig. 3a2). The Shannon index significantly changed in the 0.054 mg·m^−3^ CH_2_O group after 6 weeks (Mann–Whitney *U* test, *P* < 0.05), unlike in the other groups (Fig. 3a3). Then, we determined the patterns of bacterial communities at 6 and 12 weeks. We found that the formed bacterial communities were significantly different at both 6 (PERMANOVA based on Bray–Curtis distance, R^2^ = 0.197, *P* = 0.005) and 12 weeks (PERMANOVA based on Bray–Curtis distance, R^2^ = 0.223, *P* = 0.008) according to the CH_2_O levels (Fig. 3b1 and Supplementary Table S1). The Chao1 was significantly less in the 0.25 mg·m^−3^ CH_2_O group than the 0.054 and 0.1 mg·m^−3^ CH_2_O groups at 6 weeks (Mann–Whitney *U* test, *P* < 0.05), while the Shannon index was significantly less in the 0.25 mg·m^−3^ CH_2_O group than the 0.054 mg·m^−3^ CH_2_O group (Mann–Whitney *U* test, *P* < 0.05) (Fig. 3b). Interestingly, all the bacterial diversity indices became consistent at 12 weeks (Fig. 3b). The quite different taxonomic category of the indicated OTUs among different CH_2_O levels at 6 and 12 weeks also confirmed differed patterns of bacterial community (Supplementary Fig. S1 and S2).

### Changes of OTUs responding to CH_2_O exposure

**Figure 4:**
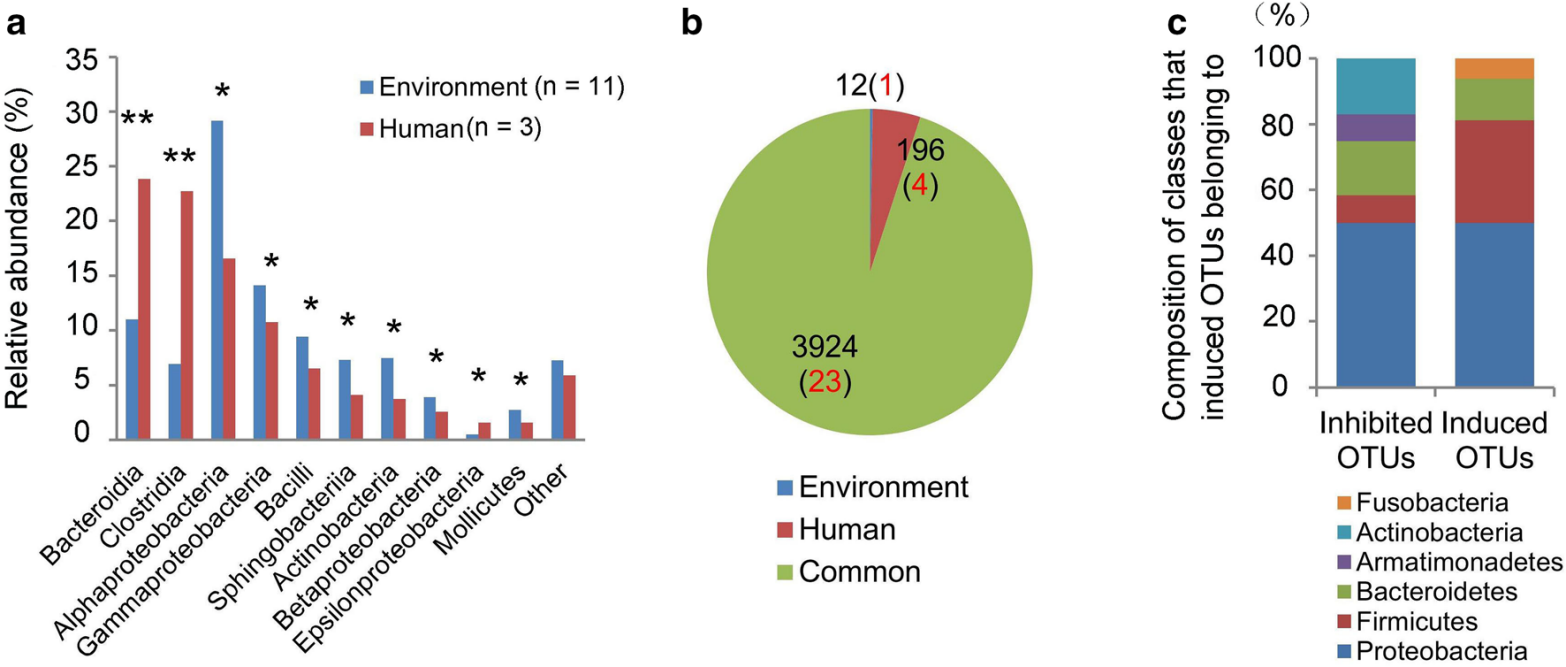
Behaviors of OTUs responding to different gaseous formaldehyde (CH_2_O) levels. The bacterial composition of environmental background and human oral cavity at the class level (**a**); the number of specific OTUs in the environmental background and human cavity (**b**); the proportion of classes that CH_2_O inhibited and induced OTUs belonging to (**c**). Numbers in red represent OTUs, which were significantly correlated with CH_2_O concentration. Mann–Whitney* U* test and Spearman’s analysis were performed. *P < 0.05, **P < 0.01.

Humans are an important source of indoor airborne microbes owing to human breath. Here, the bacterial composition at the class level (relative abundance > 1%) was quite different between the human oral and environmental background bacteria formed in an empty room over 3 months (Fig. 4a). Bacteroidia and Clostridia were significantly higher in human oral microbiota (Mann–Whitney *U* test, *P* < 0.01). In contrast, Alphaproteobacteria, Gammaproteobacteria, Bacilli, Sphingobacteriia, Actinobacteria, Betaproteobacteria, Epsilonproteobacteria, and Mollicutes were significantly higher in the environmental microbiota (Mann–Whitney *U* test, *P* < 0.05) (Fig. 4a). The number of specific OTUs in the human oral cavity and environmental background were 196 and 12, respectively; 3924 OTUs were common (Fig. 4b). We obtained 28 OTUs significantly correlated with CH_2_O concentration by the Spearman’s analysis (Table 1): four belonged to the human oral cavity, one to the environmental background (Fig. 4b). Thus, the behavior of bacteria from humans was affected by CH_2_O exposure. There were 12 inhibited OTUs and 16 induced OTUs (Table 1). We found that inhibited OTUs belong to Proteobacteria (50% of all inhibited OTUs). In comparison, induced OTUs mainly belong to Proteobacteria (50% of all induced OTUs) and Firmicutes (31% of all induced OTUs) (Fig. 4c). Thus, CH_2_O may often induce Firmicutes except for Proteobacteria.

**Table 1:** Information of gaseous formaldehyde inhibited and induced OTUs.

OTU ID	rho	*p* value	Mean of relative abundance	Phylum	Genus	Source
**Inhibited OTUs**						
OTU216	− 0.47	0.004	0.0010	Proteobacteria	*Acinetobacter*	Common
OTU174	− 0.44	0.008	0.0059	Proteobacteria	*Pseudomonas*	Common
OTU482	− 0.43	0.008	0.0007	Firmicutes	*Peptoclostridium*	Common
OTU225	− 0.41	0.013	0.0020	Proteobacteria	*Paracocccus*	Common
OTU122	− 0.39	0.018	0.0079	Armatimonadetes	*–*	Environment
OTU1416	− 0.38	0.024	0.0002	Bacteroidetes	*Terrimonas*	Common
OTU118	− 0.37	0.026	0.0005	Proteobacteria	*Vibrio*	Human
OTU531	− 0.36	0.029	0.0016	Proteobacteria	*Ralstonia*	Common
OTU1001	− 0.36	0.032	0.0005	Bacteroidetes	*Mucilaginibacter*	Common
OTU1281	− 0.35	0.034	0.0001	Proteobacteria	*Rhizomicrobium*	Common
OTU768	− 0.35	0.036	0.0003	Actinobacteria	*–*	Common
OTU880	− 0.35	0.038	0.0001	Actinobacteria	*Pseudonocardia*	Common
**Induced OTUs**						
OTU1582	0.33	0.050	0.0003	Firmicutes	*Lachnospiraceae_NK4A136_group*	Common
OTU358	0.33	0.049	0.0007	Fusobacteria	*Fusobacterium*	Human
OTU616	0.33	0.047	0.0001	Proteobacteria	*–*	Common
OTU1643	0.34	0.040	0.0003	Firmicutes	*Dorea*	Common
OTU3379	0.35	0.037	0.0001	Proteobacteria	*Sphingobium*	Common
OTU26	0.36	0.030	0.0019	Proteobacteria	*uncultured*	Common
OTU718	0.36	0.029	0.0001	Proteobacteria	*Craurococcus*	Common
OTU107	0.38	0.024	0.0017	Proteobacteria	*Acinetobacter*	Common
OTU112	0.38	0.021	0.0003	Firmicutes	*–*	Human
OTU530	0.39	0.020	0.0007	Proteobacteria	*Enterobacter*	Human
OTU875	0.40	0.017	0.0012	Firmicutes	*Lactococcus*	Common
OTU3993	0.40	0.015	0.0002	Proteobacteria	*Roseateles*	Common
OTU2366	0.40	0.014	0.0003	Firmicutes	*Granulicatella*	Common
OTU1592	0.41	0.014	0.0006	Bacteroidetes	*–*	Common
OTU1555	0.43	0.009	0.0002	Bacteroidetes	*Porphyromonas*	Common
OTU603	0.44	0.007	0.0009	Proteobacteria	*Methylobacterium*	Common

Spearman analysis was carried out.

### The hazard of CH_2_O shaped bacterial communities

**Figure 5:**
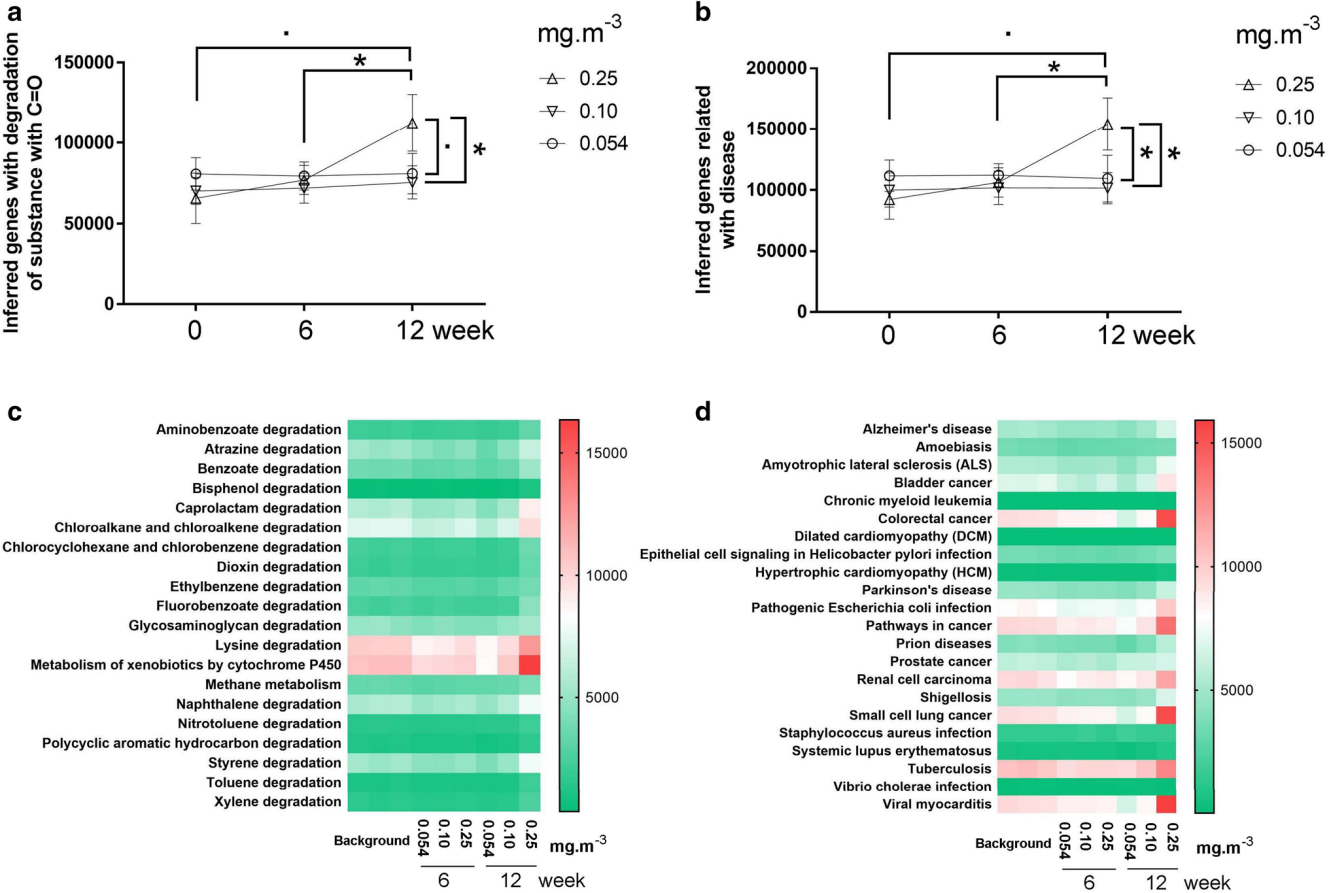
Analysis of the bacterial community's function in different formaldehyde (CH_2_O) groups using the phylogenetic investigation of communities by reconstruction of unobserved states. Inferred genes of pathways related to the degradation of substance with C=O in different CH_2_O groups at different time points (**a**); inferred genes of pathways related to disease at different time points (**b**); heat map of inferred genes of pathways related to the degradation of substance with C=O in different CH_2_O groups at different time points (**c**); heat map of inferred genes of pathways related to disease in different CH_2_O groups at different time points (**d**).

**Figure 6:**
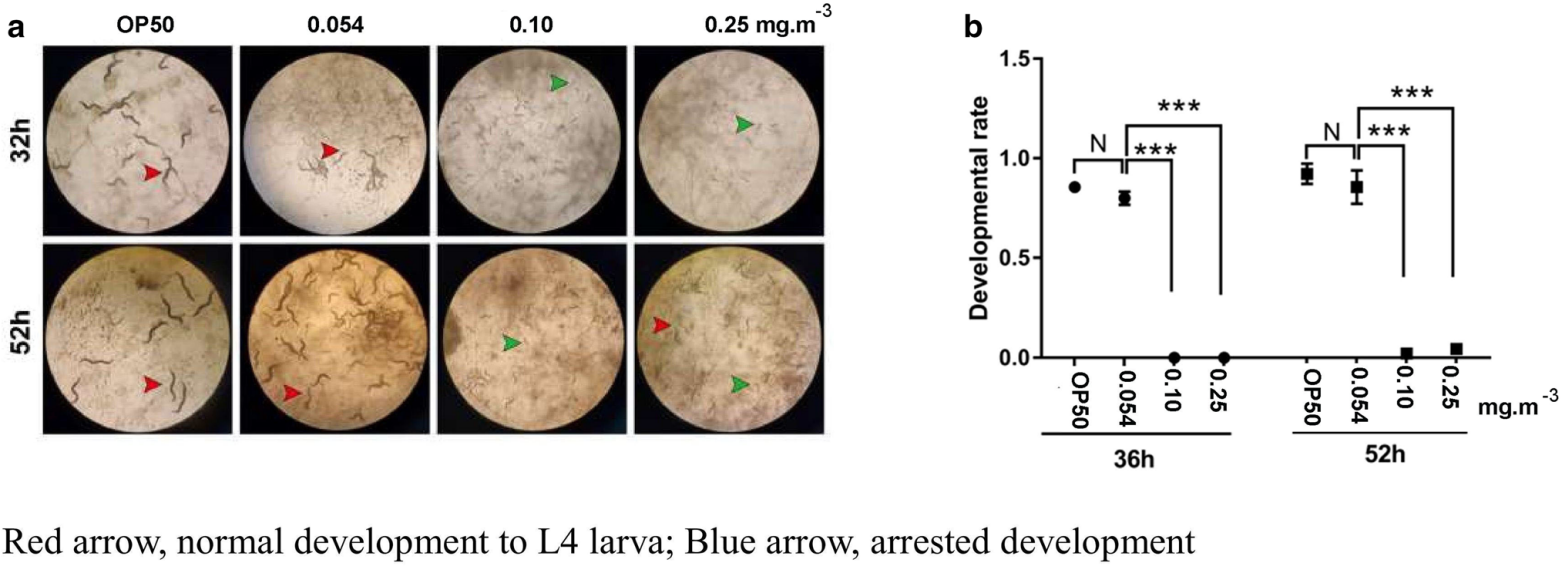
Development of *Caenorhabditis elegans* fed with bacteria collected at 12 weeks. Picture of *Caenorhabditis elegans* development (**a**); comparation of development rate fed with bacteria from differed source (**b**). Mann–Whitney* U* test was performed. *P < 0.05, **P < 0.01, ***P < 0.001.

Function forecast results showed that the inferred genes of pathways related to chemical degradation increased at 12 weeks in the 0.25 mg·m^−3^ CH_2_O group (Mann–Whitney *U* test, *P* < 0.1, *P* < 0.05) compared with the baseline (0.054 mg·m^−3^ at 0 week and 6 week) and were more than those in the 0.054 mg·m^−3^ (Mann–Whitney *U* test, *P* < 0.1) and 0.1 mg·m^−3^ CH_2_O groups (Mann–Whitney *U* test, *P* < 0.05) at 12 weeks (Fig. 5a). Similarly, the inferred genes of pathways related to disease increased at 12 weeks in the 0.25 mg·m^−3^ CH_2_O group (Mann–Whitney *U* test, *P* < 0.1, *P* < 0.05) compared with the baseline (0.054 mg·m^−3^ at 0 week and 6 week) and were significantly more than those in the 0.054 mg·m^−3^ (Mann–Whitney *U* test, *P* < 0.05) and 0.1 mg·m^−3^ CH_2_O groups (Mann–Whitney *U* test, *P* < 0.05) at 12 weeks (Fig. 5b). Detailed KEGG data showed that a higher CH_2_O level could result in a more active chemical degradation pathway (B.1 vs. B.3 and C.1 vs. C.3; Fig. 5c). Additionally, more prolonged exposure may contribute to bladder cancer, amyotrophic lateral sclerosis, Alzheimer's, and other diseases (Fig. 5d). So far, to our knowledge, there was no effective way to evaluate the hazard of one bacterial community type except by functional inferences. Thus, we attempted to use the interaction between bacteria and *Caenorhabditis elegans* to evaluate its hazard. We explored the development of *Caenorhabditis elegans* fed with bacteria collected at 12 weeks. It was found that after 32 h of exposure, 80% of nematodes were already in the young adult stage when exposed to *Escherichia coli* OP50 and C.1. In contrast, all nematodes were in the stage before L4 larva when exposed to C.2 and C.3. Similar results were observed after exposure for 52 h (Fig. 6). This finding showed that the development of *Caenorhabditis elegans* was substantially suppressed in the 0.1 mg·m^−3^ and 0.25 mg·m^−3^ CH_2_O groups, indicating a higher hazard of bacterial communities exposed to a higher CH_2_O level.

## Discussion

China's rapid modernization and urbanization have led to changes in daily living patterns and more time indoors. The issue of indoor pollution has attracted increasing attention. Many ubiquitous indoor pollutants exceed the recommended levels, including formaldehyde, benzene, other VOCs, and particulate matter^17^. Although indoor pollutants can arise from chemical, physical, and biological sources, few studies have considered the interactions among different pollutants. CH_2_O is an important precursor of many chemical compounds but poses a significant threat to human health due to its widespread use, toxicity, and volatility. Thus, we analyzed the community composition of live bacteria exposed to different CH_2_O levels.

There was a distinct difference between human respiratory gas and environmental background (Fig. 4a), suggesting inhabitant was an important source of indoor bacteria^22,23^. A significant increase of 0.054 mg·m^−3^ group in Chao1 at 6 weeks (Mann–Whitney *U* test, *P* < 0.05) (Fig. 3a2) also convinced the newer OTUs into the indoor environment after the addition of respiratory bacteria. The distinctly different bacteria between human respiratory gas and environmental background also resulted in a significant increase in the Shannon indexes (Mann–Whitney *U* test, *P* < 0.05) (Fig. 3a3). Gaseous formaldehyde is used to inactivate bacteria and B-subtilis spores at high concentration^24^. The toxin of gaseous formaldehyde in the 0.254 mg·m^−3^ concentration may explain the significantly lower value of Chao1(Mann–Whitney *U* test, *P* < 0.05), and Shannon index (Mann–Whitney *U* test, *P* < 0.05) at 6 week compared with 0.054 mg·m^−3^ concentration (Fig. 3b2,b3). Meanwhile, the indoor bacterial community structure significantly changed at 6 weeks (PERMANOVA based on Bray–Curtis distance, R^2^ = 0.192, *P* = 0.029) (Fig. 3b1 and Supplementary Table S2). The diversity indices and the relative abundance of the most abundant genera are influenced by temporal and spatial axes^25^. In this study, the exposure period is an important factor. Thus, we also monitored the bacterial community at 12 week. Interestingly, the value of Chao1 in the 0.254 mg·m^−3^ concentration increased at 12 week (Fig. 3b2). This was possibly due to some taxa's adaption to higher CH_2_O levels. So far, many formaldehyde-resistant bacteria were identified in seawater^26^, river sediment^27^, including *Pseudomonas putida*^28^, *Paracoccus *sp*. FD3*^29^, *Bacillus *sp*. BZ-001H*^30^, *Rhodobacter sphaeroides*^31^, *acetic acid bacteria*^32^, *Escherichia coli VU3695*^33^, *Halomonas *sp*. MAC*^33^, *Methylobacterium *sp*.*^34^, and *Sphingomonas *sp*.*^35^. Indicated OTUs belonging to *Methylobacterium* in the 0.25 mg·m^−3^ CH_2_O group at 12 week might explain some bacteria's formaldehyde-resistant characteristic (Supplementary Fig. S1).

The CH_2_O injection did not change the total DNA content of samples (Supplementary Fig. S3), possibly due to limited mass propagation nutrients. The bactericidal action of CH_2_O is not as apparent as in a solution. The bacterial community structure was examined, and 16 CH_2_O induced OTUs were found. CH_2_O-induced OTUs mostly came from the indoor environment (13/16) (Table 1), which showed that some indoor bacteria had a strong plasticity response to gaseous CH_2_O exposure. The bacteria's response from the human to gaseous CH_2_O exposure could also affect the indoor bacterial composition (3/16) (Table 1). Unfortunately, the function of most OTUs was unclear. Remarkably, CH_2_O-induced OTU603 belongs to *Methylobacterium* (Table 1). Microorganisms belonging to *Methylobacterium* are ubiquitous facultative methylotrophic Gram-negative rods that can degrade CH_2_O^34^. CH_2_O-induced OTU3993 belongs to the *Roseateles.* Although there was no evidence showing *Roseateles* could degrade CH_2_O, its degradation of aliphatic and aliphatic–aromatic copolyesters has been proved^36,37^, which possibly indicates its ability to degrade CH_2_O. OTU603 and OTU3993 may be considered to monitor indoor long-term CH_2_O pollution. Meanwhile, they would perhaps be used to evaluate the bacterial community's health risk in CH_2_O pollution due to its significant positive correlation with many disease processes (Supplementary Table S3).

Indoor CH_2_O pollution is a severe issue that can result in various diseases. We can decrease the indoor level of CH_2_O by increasing ventilation in our daily lives. However, this cannot eliminate the bacteria attached to indoor surfaces, forming bacterial communities in a polluted indoor environment. The remaining bacterial communities in the room may enter the air accompanied by human activities. Bacteria enter the intestinal tract and lungs through ingestion and respiration. Many studies have shown that an imbalance in the bacterial community in these organs correlates with type 1 diabetes^38–40^. Here PICRUSt2 was used to infer the function of bacterial communities in each sample. The inferred genes of pathways related to chemical degradation increased in the 0.25 mg·m^−3^ CH_2_O group at 12 weeks. This was possible because CH_2_O acts as a carbon source for bacteria and enrichment of degradation-related pathways. The function forecast and test of the development of *Caenorhabditis elegans* fed with bacteria collected at 12 weeks emphasized the more significant hazard of the bacterial community in higher CH_2_O level conditions with 12 weeks of exposure (Fig. 6). Studies showed a positive correlation between CH_2_O exposure and the development of leukemia, particularly myeloid leukemia^41,42^. However, despite studies investigating the molecular mechanism of CH_2_O toxicity in animals and cell lines, the underlying mechanism remains unclear^43–47^. Furthermore, several inhalation studies have not detected DNA adducts outside the nasal tissues of rats or monkeys^48^. This study revealed a strong relationship between CH_2_O exposure and the indoor bacterial community. CH_2_O and indoor microbes possibly work together in disease development.

The indoor environment fluctuates with the outdoor environment and inhabitants' activities^49–52^, which can mask the relationship of interest. Therefore, we eliminated the outdoor environment's effects and human activity in our experimental design, retaining only the human bacterial input from one person. Our findings demonstrate that the interaction between CH_2_O and indoor bacteria (including the human input) could not be neglected when studying the indoor environment. CH_2_O levels and exposure time were vital factors shaping the indoor bacterial community. The indoor environment is complex and disturbed by the outdoor environment, human behavior, pets, and more VOCs. Thus, further research is required to explore the relationship between indoor pollutants, indoor microorganisms, and human health. This study provides a basis for future research on the interaction between indoor pollutants and the bacterial community structure, which will improve our understanding of the effects of indoor pollution on human health.

### Limitations of this study

The indoor microbes are complex, comprised of bacteria, fungi, archaea. Here, we only explored the variation of bacteria exposed to CH_2_O. Although the most abundant microbes are bacteria, there may be a possible effect of CH_2_O on other microbes. Meanwhile, indoor bacterial communities are affected by many human activities, including breathing, cough, walking, and source from the human nasal cavity, skin, oral cavity, hair, fomites. Human microbiota varies among humans, making the indoor source of microbes more complex. There are many indoor VOCs, and different emitting characteristics make the indoor environment more complex. Our experiments simplified the indoor environment and building structure, which was different from the actual indoor environment. Furthermore, the limited containers and sampling time points made us come to a relatively conservative conclusion. The size of the experimental chambers in this study is far smaller than the actual living space and we ignored the effect of ventilation and airflow on formaldehyde diffusion and bacterial distribution. A simulation cabin with precise control of temperature, humidity, light, airflow, and microorganism input would be useful to explore the complex interaction in the indoor environment.

## Conclusion

This study used individual containers to mimic daily living conditions, allowing us to investigate the relationship between gaseous CH_2_O and indoor bacteria. CH_2_O levels and exposure time affected the bacterial community structure. The trend of Chao1, and Shannon index were different among varied CH_2_O levels. CH_2_O-induced OTUs coming from the indoor environment and humans mainly belong to the Proteobacteria and Firmicutes. Longer and higher CH_2_O exposure environments may form bacterial communities with a greater hazard to human health.

## Methods

### Indoor microbial environment formation in containers

In this study, 20-L glass containers were used to simulate the indoor environment (Fig. 1a). Each container had four holes on the top (two with a 24-mm diameter and two with a 40-mm diameter) and one hole in the front (hole T: 15-cm diameter). The background reading of formaldehyde was about 0.02 mg·m^−3^. The room is located on the top floor of one three-floor building surrounding residential buildings. The selected empty room was used as an office room with one person staying in it for three days (3–6 h/day) a week for 2 months. Then, the room stood empty for a month without habitation until the research started. We placed 15 containers disinfected with 75% ethanol in the room marked as 12 W (Fig. 1b). All containers were placed on one side of the room to avoid sunlight. Three sterile clean 90-mm Petri-dishes (without agar) were placed inside each container through the hole T. All holes were opened, and the containers were left open for 3 months to replicate the normal indoor environment. Indoor microbial sources varied, including outdoor air and soil. Thus, within 3 months, one window was left open to permit natural ventilation to make the container's internal environment comprised various microorganisms and environmental substances similar to the indoor environment without occupants. After 3 months, we entered the room wearing lab gowns, masks, and gloves to perform the first sampling by smearing a 3 × 3 cm area on one 90-mm dish in the container using sterile swabs dipped in 100 μL of sterile normal saline.

### CH_2_O exposure operation

Then, we blocked all holes [one for CH_2_O injection (hole A), one for human input (hole B), one for ventilation (hole C), and one for sampling (hole D)] to ensure minimum contamination (Fig. 1a). Hole C, which was intended to simulate natural ventilation, was blocked with sterile cotton to prevent microbial circulation, and hole B for human input was blocked with a glass valve that could maintain the passage of the human respiratory gas by adjustment. When oral microbes were added by oral respiration, the glass valve was switched on. Hole A and D were blocked with stoppers and opened during the exposure procedure. Hole T was covered with powder-free latex gloves to ease inflation and keep the container airtight when respiratory gas was imported. Hole T was blocked until the research ended. We began the exposure experiment after blocking all holes. On the first day, the breath of a person about 2–3 L respiratory gas was blown into each container at 08:00 h, and 0.5–1 L of respiratory gas was blown into 30-mL sterilized saline water in a tube. Then, the collection tube was stored at 4 °C. On the same day, different volumes of CH_2_O were injected at 17:00 h to obtain final CH_2_O levels of 0.054, 0.1, and 0.25 mg·m^−3^, confirmed by Interscan 4160-1999b (Interscan, USA) 1 h after injection in a separate trial (Supplementary Fig. S4a). Hole C was covered with a preservative film to maintain impermeability on the first day. The preservative film was removed on the second day and absorbent cotton was placed in hole C at 08:00 h to allow CH_2_O release to simulate natural indoor ventilation. On the third day, an operation identical to that performed on the first day was conducted. This exposure cycle was repeated every two days (Supplementary Fig. S5). Samples were collected at 6 and 12 weeks by smearing a 3 × 3 cm area on unsampled 90-mm dishes, respectively, using sterile swabs dipped in 100 μL of sterile normal saline, then storing these in individual sterile 2-mL tubes. Fifteen containers were used in this research (five containers for each CH_2_O level). One was a human oral bacterial source to exclude person-to-person differences. Further, 0.5–1 L of respiratory gas was blown into a 50-mL collection tube with 30 mL sterilized saline was used to collect the oral bacteria after oral bacterial input. Collections for 4 weeks were combined as one human oral bacterial sample. Three human oral bacterial samples were obtained for analysis. We conducted a separate trial to monitor the CH_2_O level in the container for 24 h after removing the preservative film in hole C and keeping absorbent cotton after CH_2_O injection by Interscan 4160-1999b (Interscan). It was confirmed that 24-h volatilization was long enough to recover the CH_2_O level to baseline (Supplementary Fig. S4b).

### Final sampling

When the experiment ended, 50-mL sterile saline water was used to wash the container, and the solution was collected. The solution was centrifuged at 10,000×*g* for 15 min; the supernatant was discharged and 10-mL sterile saline water was used to resuspend the pellet; centrifuged at 10,000×*g* for 15 min; discharged the supernatant and added 2-mL sterile saline water to resuspend the pellet to make a final solution. Next, a 200-µL solution was used to coat the surface of normal nematode growth medium (NGM) plates to feed wild-type *Caenorhabditis elegans* N_2_ nematodes to monitor its development.

We conducted three exposed CH_2_O levels, and the experimental operation and grouping are shown in Fig. 1b and Supplementary Table S4. In the experiment, the container's air disturbance occurred in the operation of human bacterial input and the discharge of CH_2_O, facilitating an exchange of microbes between air and surface. Thus, we used surface samples on behalf of airborne microbes. The temperature scale and relative humidity during the experiment (0–12 week) were 20.5–36.5 °C and 14–63%, respectively. The experiments were conducted under the approval of the Peking Union Medical College (PUMC) (approval nos. JS-2548). All the methods were performed following relevant guidelines and regulations of PUMC. Informed consent was obtained from all participants.

### DNA extraction, polymerase chain reaction (PCR), and next-generation sequencing

PMAxx (Biotium, Inc., Fremont, CA, USA) was used to determine the bacterial community structure of live bacteria. PMAxx is a photoreactive dye that binds to dsDNA with a high affinity. Upon photolysis with visible light, the PMAxx dye becomes covalently attached to dsDNA. The PMAxx-modified dsDNA cannot be amplified by PCR. Thus, in a population of live and dead cells, only dead cells are susceptible to DNA modification due to compromised cell membranes. This unique feature makes PMAxx highly useful in the selective detection of live bacteria^53–55^. Upon completion of sampling, 500-μL sterile normal saline was added to each tube, and the sample was vortexed for 10 min. Then, the swab was discarded, and 5 μL of a 2.5 mM working solution of PMAxx dye was added to the 500-μL sample. The tubes were incubated in the dark for 5–10 min at room temperature and then exposed to light (465–475 nm) for 30 min to crosslink PMAxx with DNA. This photoreactive dye becomes covalently attached to the double-stranded DNA (dsDNA) with a high affinity upon photolysis with visible light. The resulting PMAxx-modified dsDNA cannot be amplified. Because only dead cells are susceptible to this DNA modification due to their compromised cell membranes, PMAxx is highly useful for the selective detection of live bacteria^53–55^. Precipitates were obtained by centrifugation at 12,000×*g* for DNA extraction using the PowerSoil DNA Isolation Kit (MO BIO Laboratories, Inc., Carlsbad, CA, USA) according to the manufacturer’s recommendations. The extracted DNA was diluted to 1 ng/μL and stored at − 20 °C until further processing. 16S metagenomic sequencing library was prepared according to the protocol of Illumina. The diluted DNA was used as a template for PCR amplification (26 cycles: 94 °C 30 s, 56 °C 30 s, and 72 °C 30 s) of the bacterial 16S rRNA gene with the V3–4 variable regions universal primers 343F (TACGGRAGGCAGCAG) and 798R (AGGGTATCTAATCCT) linked with overhand adapter^56,57^ and the HiFi Hot Start Ready Mix (KAPA, Roche, USA). Negative control was used in the same amplification system using sterile deionized water as the template. Amplicon quality was visualized using gel electrophoresis. The amplicons were purified using the AxyPrep DNA Gel Extraction Kit (Axygen Biosciences, Union City, CA, USA). The purified amplicons were attached indices and Illumina sequencing adapters by amplified with primers of overhang adapters (7 cycles), and purified again with AMPure XP beads. The batch with no visible 400-bp band of negative control was treated using the following steps. The final amplicon level was then quantified using the Qubit dsDNA Assay Kit (Promega, USA). An equal number of the purified amplicons was pooled for subsequent sequencing using the MiSeq Sequencing System (Illumina, Inc., San Diego, CA, USA).

### Sequence processing

Raw sequencing data were stored in FASTQ format. Paired-end reads were preprocessed using the Trimmomatic software^58^ to detect and cut off ambiguous bases (N) and low-quality sequences with an average quality score of < 20 using the sliding window-trimming approach. After trimming, paired-end reads were assembled using the FLASH software^59^. The assembly had the following parameters: 10 bp of minimal overlap, 200 bp of maximum overlap, and 20% maximum mismatch rate. Further denoizing of the sequences involved (1) abandoning reads that had ambiguous, homologous sequences or were < 200 bp, but retaining reads with 75% of bases above Q20, and (2) for detecting and removing any chimeric reads using the QIIME software (v.1.8.0)^60^.

Clean reads were subjected to primer sequence removal and clustering to generate operational taxonomic units (OTUs) using the UPARSE software with a 97% similarity cutoff^61^. The representative read of each OTU was then selected using the QIIME package. All representative reads were annotated and blasted against the Silva database v.123 (16S rDNA) using the ribosomal database classifier (confidence threshold = 70%)^62^. Forty-five samples were collected from different containers at 0, 6, and 12 weeks. Any abnormal samples that had been disturbed during the experiment were disposed of, leaving 36 environmental samples and three human samples (Supplementary Table S4). The dataset comprised 32,734 ± 3231 (mean ± standard deviation) valid reads per sample, clustered into 4132 different OTUs. Each sample was rarefied to 25,740 sequences, and the coverage of the samples was > 0.99 (Supplementary Fig. S6). The Chao1 richness estimator and Shannon index were calculated using the QIIME package. The phylogenetic investigation of communities by reconstruction of unobserved states 2 (PICRUSt2) was used to predict the KEGG category based on 16Sr DNA of samples^63^.

### *Caenorhabditis elegans* strains: maintenance and development assay

Indoor airborne bacteria directly affect the function of respiratory epithelial cells. We selected wild-type *Caenorhabditis elegans* N_2_ nematode fed with bacteria as our animal model to evaluate bacteria's health risk. Nematodes were maintained on NGM plates seeded with *Escherichia coli* OP50 as a food source at 25 °C as described previously^64^. Gravid nematodes were lysed with a bleaching mixture (0.45 M NaOH and 2% HOCl) to separate eggs from animals. The collected eggs were allowed to develop into synchronous L1 larvae. Aged synchronous L1 larvae were transferred to NGM plates containing *Escherichia coli* OP50 or bacterial samples collected from groups C.1, C.2, and C.3 at 12 weeks and raised for 52 h at 25 °C. Each group comprised three replicates with 30 synchronous L1 larvae each. Visual scoring of nematode development was performed after feeding for 36 and 52 h.

### Statistical analyses

In this experiment, group A.1, A.2, and A.3 were considered environmental backgrounds without occupants. Latex gloves blocked the front hole of the container. During exposure, broken latex gloves disturbed indoor air composition. Thus, disturbed samples were removed. Further, the Chao1 value of samples in each group was analyzed by a boxplot. A sample with an outlier (< Q1–1.5 interquartile range or > Q3 + 1.5 interquartile range) was eliminated to ensure the exclusion of potential bacterial contamination during CH_2_O exposure. PcoA analysis were conducted based on Bray–Curtis distance. Permutational multivariate analysis of variance (PERMANOVA) was performed to compare bacterial community differences between the two groups based on Bray–Curtis distance using the package “Vegan”^65^ in R. Mann–Whitney *U* test was conducted to compare the abundance of OTUs, classes, and diversity index between different groups using the package “State”^66^ in R. *P*-values of < 0.05 indicated a statistically significant difference for PERMANOVA and Mann–Whitney *U* test. The significantly higher OTUs in the environmental background samples were regarded as environment-specific OTUs; the significantly higher OTUs in human samples were considered human-specific OTUs. Spearman analysis was used to explore the correlations. The correlations between the abundance of OTUs and CH_2_O exposure concentration were analyzed. The significantly negatively correlated OTUs were inhibited OTUs, and significantly positively correlated OTUs were induced OTUs. The correlations between the abundance of induced OTUs and matched reads related to degradation and disease pathways were also analyzed. A heatmap was generated using the package “Pheatmap”^67^ in R environment using raw data.

### Nucleotide sequence accession number

All bacterial 16S rRNA gene sequences generated in this study are deposited in the National Center for Biotechnology Information Sequence Read Archive (http://www.ncbi.nlm.nih.gov/sra) under the accession number SRP158743.

## Supplementary Information

Supplementary Information.
