# Supplementary material for: Effect of formaldehyde exposure on bacterial communities in simulating indoor environments
Source: Sci Rep. 2021 Oct 18;11:20575. doi: 10.1038/s41598-021-00197-7 (PMC8523742; doi:10.1038/s41598-021-00197-7)
Supplement: Supplementary file 1 — Supplementary Information. [file 41598_2021_197_MOESM1_ESM.docx]

**Supplementary for:**

**Effect of formaldehyde exposure on bacterial communities in simulated indoor environments**

Jianguo Guo^1,2^, Yi Xiong^3^, Taisheng Kang^1,2^, Hua Zhu^1,2^, Qiwen Yang^4^,Chuan Qin^1,2*^

1 NHC Key Laboratory of Human Disease Comparative Medicine, Institute of Laboratory Animal Sciences, CAMS&PUMC, Beijing 100021, China

2 Key Laboratory of Human Diseases Animal Model, State Administration of Traditional Chinese Medicine, Beijing 100021, China

3 Department of Food Science and Engineering, School of Chemistry and Chemical Engineering, Harbin Institute of Technology, Harbin 150001, China

4 Department of Clinical Laboratory, Peking Union Medical College Hospital, Peking Union Medical College, Chinese Academy of Medical Sciences, Beijing 100730, China.

Corresponding author: Chuan Qin

E-mail: qinchuan@pumc.edu.cn

Postal address: Pan Jia Yuan Nan Li No. 5, Chao Yang District, Beijing 100021, China.

Tel: +86-010- 87778141

Fax number: +86-010 67761943


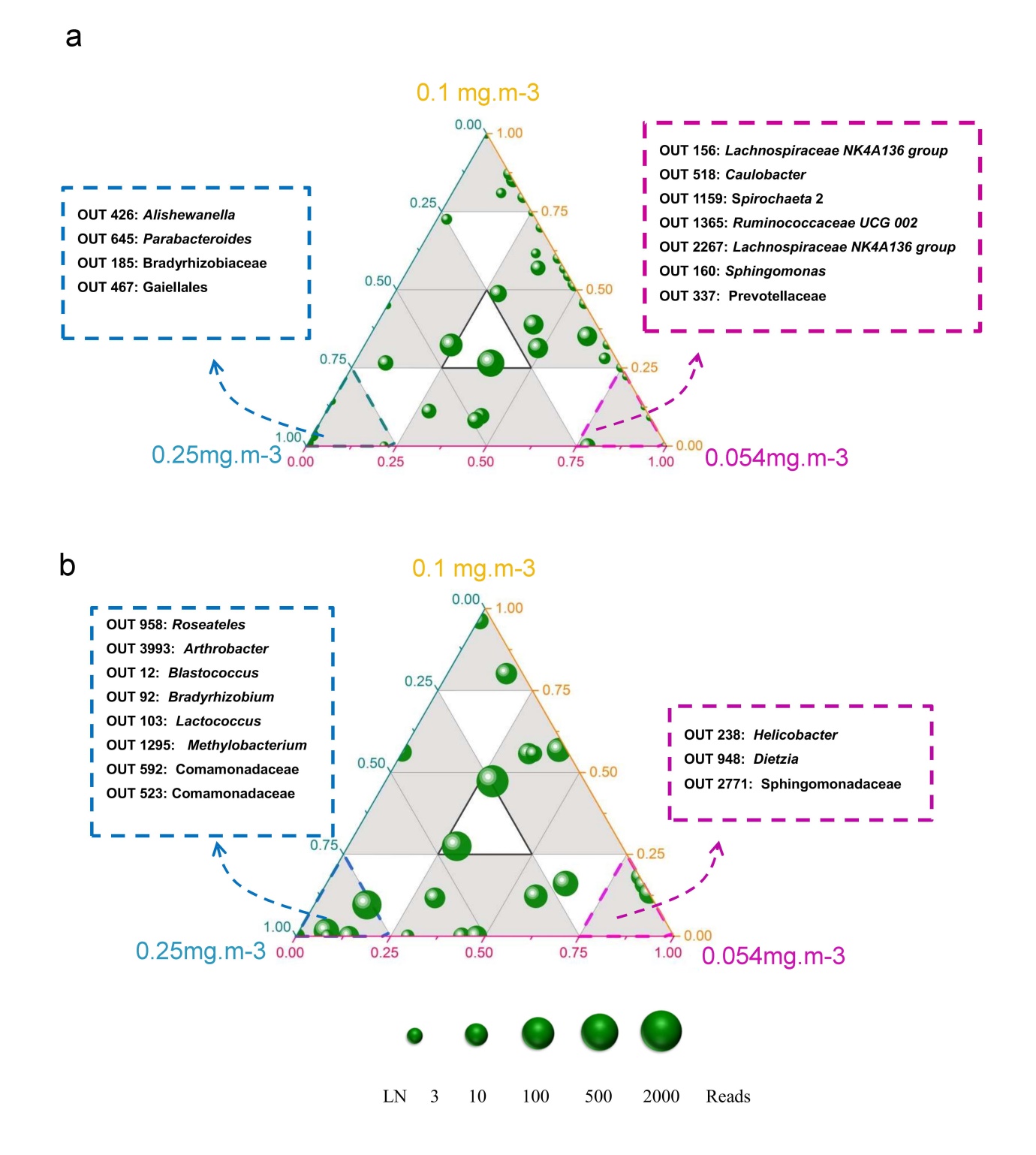


**Figure S1** The distribution of OTUs in the three CH_2_O levels at 6 and 12 weeks. The significantly different OTUs are shown. The OTU, whose proportion in the ternary diagram was >0.75 in any group at these periods, was considered the group's indicated OTU.

**Figure. S2** Proportion of the classes of indicated OTUs belong to the 0.054 and 0.25 mg.m^-3^ CH_2_O groups at 6 and 12 weeks


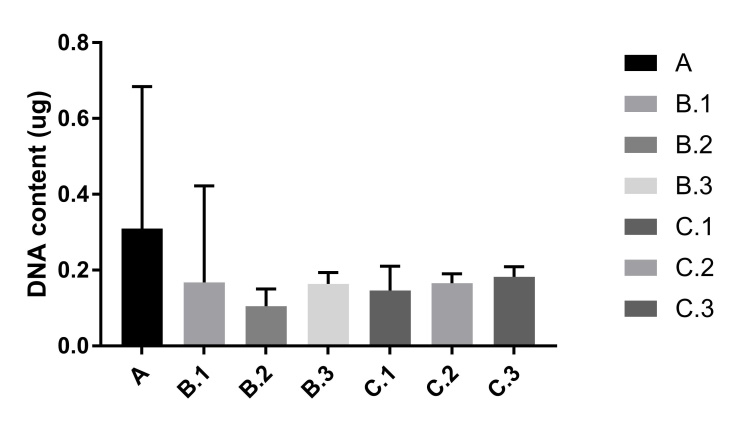


**Figure. S3** Content of DNA in samples

| a |
| --- |
|  |
| b |
|  |

**Figure. S4** Formaldehyde (CH_2_O) concentration in the container over time after CH_2_O injection (a) and trend of CH_2_O concentration after removal of the preservative film and retaining of the absorbent cotton in the hole (b)


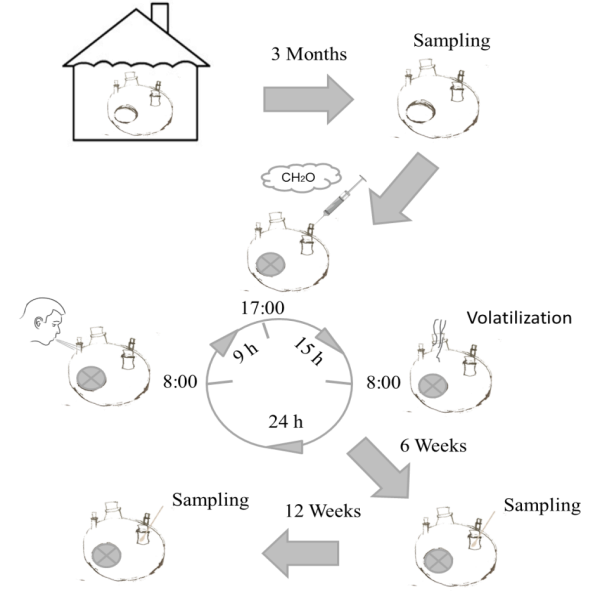


**Figure. S5** Schematic of experimental operation


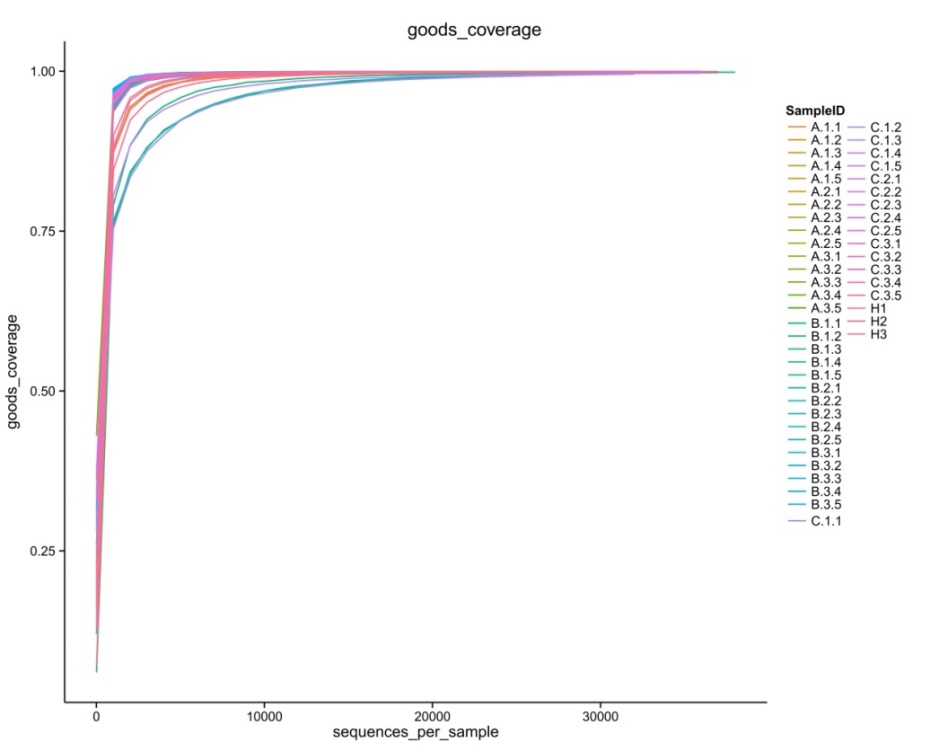


**Figure. S6** Coverage of the sequencing samples

**Table S1** PERMANOVA analysis of bacterial communities between different TREATs at the same exposure time

|  |  | Bray-Curtis distance | |  | Jaccard distance | |
| --- | --- | --- | --- | --- | --- | --- |
|  |  | R^2^ | *P* |  | R^2^ | *P* |
| Comparisons of two groups | A.1 vs A.2 | 0.172 | 0.327 |  | 0.168 | 0.412 |
|  | A.1 vs A.3 | 0.143 | 0.454 |  | 0.143 | 0.511 |
|  | A.2 vs A.3 | 0.180 | 0.275 |  | 0.174 | 0.282 |
|  |  |  |  |  |  |  |
|  | B.1 vs B.2 | 0.169 | 0.082 |  | 0.161 | 0.089 |
|  | B.1 vs B.3 | 0.144 | 0.040* |  | 0.138 | 0.040* |
|  | B.2 vs B.3 | 0.148 | 0.027* |  | 0.139 | 0.034* |
|  |  |  |  |  |  |  |
|  | C.1 vs C.2 | 0.150 | 0.294 |  | 0.148 | 0.295 |
|  | C.1 vs C.3 | 0.196 | 0.157 |  | 0.186 | 0.174 |
|  | C.2 vs C.3 | 0.181 | 0.010* |  | 0.162 | 0.004** |
|  |  |  |  |  |  |  |
| Comparisons according to CH_2_O levels | A.1, A.2, A.3 | 0.208 | 0.22 |  | 0.203 | 0.357 |
|  | B.1, B.2, B.3 | 0.197 | 0.005** |  | 0.185 | 0.008** |
|  | C.1, C.2, C.3 | 0.223 | 0.008** |  | 0.209 | 0.011* |

“*”: *P* < 0.05, “**”: *P* < 0.01, “***”: *P* < 0.001

**Table S2** PERMANOVA analysis of bacterial communities between different CH_2_O exposure time in the same TREAT

|  | Bray-Curtis distance | |  | Jaccard distance | |
| --- | --- | --- | --- | --- | --- |
|  | R^2^ | *P* |  | R^2^ | *P* |
| A.1 vs B.1 | 0.192 | 0.029* |  | 0.176 | 0.022* |
| B.1 vs C.1 | 0.198 | 0.026* |  | 0.188 | 0.032* |
| A.1 vs C.1 | 0.215 | 0.026* |  | 0.199 | 0.030* |
|  |  |  |  |  |  |
| A.2 vs B.2 | 0.175 | 0.393 |  | 0.172 | 0.386 |
| B.2 vs C.2 | 0.133 | 0.332 |  | 0.130 | 0.322 |
| A.2 vs C.2 | 0.146 | 0.395 |  | 0.144 | 0.428 |
|  |  |  |  |  |  |
| A.3 vs B.3 | 0.106 | 0.993 |  | 0.112 | 0.994 |
| B.3 vs C.3 | 0.156 | 0.055 |  | 0.145 | 0.056 |
| A.3 vs C.3 | 0.189 | 0.079 |  | 0.173 | 0.073 |

“*”: *P* < 0.05, “**”: *P* < 0.01, “***”: *P* < 0.001

**Table S3** Correlation between formaldehyde induced OTUs and matched reads related with degradation and diseases

|  | KEGG level 3 | **OTU**  **1582** | **OTU**  **358** | **OTU**  **616** | **OTU**  **1643** | **OTU**  **3379** | **OTU**  **26** | **OTU**  **718** | **OTU**  **107** | **OTU**  **112** | **OTU**  **530** | **OTU**  **875** | **OTU**  **3993** | **OTU**  **2366** | **OTU**  **1592** | **OTU**  **1555** | **OTU**  **603** |
| --- | --- | --- | --- | --- | --- | --- | --- | --- | --- | --- | --- | --- | --- | --- | --- | --- | --- |
| Matched reads related with degradation of substance with C=O | Aminobenzoate degradation | 0.063 | 0.191 | 0.181 | 0.113 | 0.281 | 0.055 | 0.017 | 0.004 | 0.282 | -0.129 | 0.064 | 0.435** | 0.129 | 0.196 | 0.078 | 0.375* |
|  | Atrazine degradation | 0.079 | 0.017 | 0.071 | -0.113 | 0.137 | 0.048 | -0.05 | -0.157 | 0.195 | -0.281 | 0.031 | 0.312 | 0.003 | 0.099 | 0.011 | 0.293 |
|  | Benzoate degradation | 0.054 | 0.158 | 0.131 | 0.037 | 0.19 | -0.014 | -0.031 | -0.065 | 0.229 | -0.188 | 0.014 | 0.412* | 0.13 | 0.199 | 0.029 | 0.342* |
|  | Bisphenol degradation | 0.391* | 0.122 | 0.036 | 0.191 | 0.425** | 0.203 | 0.29 | 0.09 | 0.322 | 0.091 | -0.061 | 0.443** | 0.018 | 0.012 | 0.219 | 0.482** |
|  | Caprolactam degradation | 0.097 | 0.111 | 0.098 | 0.037 | 0.223 | 0.053 | 0.016 | -0.034 | 0.264 | -0.194 | 0.051 | 0.389* | 0.087 | 0.188 | 0.066 | 0.331* |
|  | Chloroalkane and chloroalkene degradation | 0.081 | 0.171 | 0.135 | 0.056 | 0.206 | 0.072 | 0.044 | -0.06 | 0.245 | -0.143 | 0.078 | 0.389* | 0.112 | 0.248 | 0.089 | 0.318 |
|  | Chlorocyclohexane and chlorobenzene degradation | 0.011 | 0.133 | 0.152 | -0.021 | 0.198 | 0.113 | 0.017 | -0.127 | 0.164 | -0.135 | 0.038 | 0.404* | 0.016 | 0.147 | 0.088 | 0.32 |
|  | Dioxin degradation | 0.108 | 0.19 | 0.126 | 0.028 | 0.253 | 0.094 | -0.044 | 0.14 | 0.364* | -0.103 | 0.082 | 0.404* | 0.162 | 0.23 | 0.058 | 0.408* |
|  | Ethylbenzene degradation | -0.025 | 0.206 | 0.154 | 0.117 | 0.286 | -0.076 | 0.1 | 0.03 | 0.329* | -0.044 | -0.002 | 0.416* | 0.164 | 0.084 | 0.101 | 0.319 |
|  | Fluorobenzoate degradation | 0.084 | 0.196 | 0.188 | 0.165 | 0.329* | 0.209 | -0.018 | 0.065 | 0.29 | -0.06 | -0.152 | 0.466** | 0.081 | 0.06 | 0.105 | 0.401* |
|  | Glycosaminoglycan degradation | 0.003 | 0.111 | 0.137 | 0.083 | 0.182 | -0.14 | 0.166 | -0.244 | -0.014 | 0.047 | -0.07 | 0.383* | 0.048 | -0.086 | -0.05 | 0.23 |
|  | Lysine degradation | 0.062 | 0.209 | 0.132 | 0.034 | 0.216 | -0.022 | 0.004 | -0.041 | 0.252 | -0.152 | 0.07 | 0.404* | 0.145 | 0.192 | 0.021 | 0.376* |
|  | Metabolism of xenobiotics by cytochrome P450 | 0.061 | 0.186 | 0.194 | 0.026 | 0.294 | 0.08 | 0.016 | -0.024 | 0.268 | -0.069 | -0.02 | 0.435** | 0.109 | 0.158 | 0.072 | 0.393* |
|  | Methane metabolism | 0.031 | 0.159 | 0.172 | 0.129 | 0.248 | -0.071 | 0.157 | -0.074 | 0.234 | 0.013 | 0.006 | 0.42* | 0.112 | 0.06 | 0.024 | 0.431** |
|  | Naphthalene degradation | 0.087 | 0.186 | 0.151 | 0.116 | 0.254 | 0.075 | 0.136 | 0.004 | 0.285 | -0.074 | 0.012 | 0.419* | 0.143 | 0.161 | 0.096 | 0.364* |
|  | Nitrotoluene degradation | 0.059 | 0.106 | 0.136 | 0.024 | 0.208 | 0.077 | 0.196 | 0.067 | 0.256 | 0.224 | 0.009 | 0.366* | 0.126 | 0.133 | 0.009 | 0.378* |
|  | Polycyclic aromatic hydrocarbon degradation | 0.048 | 0.148 | 0.137 | -0.021 | 0.133 | 0.073 | -0.064 | -0.214 | 0.109 | -0.161 | 0.077 | 0.409* | 0.096 | 0.216 | 0.034 | 0.319 |
|  | Styrene degradation | 0.046 | 0.153 | 0.138 | -0.022 | 0.217 | 0.036 | -0.023 | -0.046 | 0.23 | -0.173 | 0.027 | 0.396* | 0.039 | 0.137 | 0.04 | 0.291 |
|  | Toluene degradation | -0.026 | 0.206 | 0.162 | 0.001 | 0.223 | 0.059 | 0.012 | 0.01 | 0.147 | -0.047 | 0.025 | 0.428** | 0.057 | 0.119 | 0.055 | 0.289 |
|  | Xylene degradation | 0.091 | 0.175 | 0.119 | -0.009 | 0.279 | 0.128 | -0.04 | 0.042 | 0.247 | -0.114 | 0.037 | 0.42* | 0.075 | 0.149 | 0.064 | 0.325 |
|  |  |  |  |  |  |  |  |  |  |  |  |  |  |  |  |  |  |
| Matched reads related with diseases | Alzheimer's disease | 0.015 | 0.201 | 0.21 | 0.025 | 0.289 | -0.045 | 0.063 | -0.11 | 0.218 | -0.113 | -0.031 | 0.482** | 0.114 | 0.02 | -0.009 | 0.404* |
|  | Amoebiasis | -0.028 | 0.015 | -0.054 | 0.02 | 0.065 | 0.231 | 0.131 | -0.235 | 0.102 | -0.07 | -0.034 | 0.181 | -0.06 | -0.024 | 0.059 | 0.275 |
|  | Amyotrophic lateral sclerosis (ALS) | 0.017 | 0.237 | 0.144 | 0.014 | 0.297 | -0.028 | 0.045 | -0.068 | 0.231 | -0.15 | -0.028 | 0.458** | 0.057 | 0.002 | 0.002 | 0.408* |
|  | Bladder cancer | 0.044 | 0.016 | 0.132 | -0.062 | 0.162 | 0.008 | -0.071 | -0.006 | 0.276 | -0.14 | 0.075 | 0.306 | 0.044 | 0.102 | 0.01 | 0.296 |
|  | Chronic myeloid leukemia | -0.098 | -0.178 | -0.084 | 0.17 | -0.284 | 0.007 | -0.173 | 0.013 | 0.02 | -0.366* | -0.065 | -0.101 | 0.056 | -0.148 | -0.227 | -0.1 |
|  | Colorectal cancer | 0.13 | 0.114 | 0.162 | -0.08 | 0.285 | 0.051 | -0.048 | -0.1 | 0.184 | -0.138 | -0.084 | 0.443** | 0.01 | 0.019 | 0.009 | 0.38* |
|  | Dilated cardiomyopathy (DCM) | 0.18 | -0.113 | 0.011 | -0.026 | 0.07 | -0.047 | 0.272 | -0.053 | -0.106 | 0.452** | -0.014 | 0.153 | 0.002 | -0.11 | 0.122 | 0.2 |
|  | Epithelial cell signaling in Helicobacter pylori infection | 0.023 | 0.153 | 0.056 | 0.111 | 0.258 | -0.09 | 0.179 | -0.116 | 0.23 | -0.029 | -0.074 | 0.419* | 0.071 | -0.132 | -0.009 | 0.459** |
|  | Hypertrophic cardiomyopathy (HCM) | 0.008 | 0.288 | 0.169 | -0.081 | 0.481** | -0.003 | 0.203 | 0.179 | 0.174 | 0.299 | 0.048 | 0.374* | 0.089 | 0.108 | 0.171 | 0.249 |
|  | Parkinson's disease | 0.041 | 0.169 | 0.185 | 0.082 | 0.264 | 0.007 | 0.117 | -0.077 | 0.212 | -0.067 | -0.054 | 0.435** | 0.073 | 0.03 | 0.045 | 0.421* |
|  | Pathogenic Escherichia coli infection | 0.04 | 0.199 | 0.194 | 0.03 | 0.292 | -0.013 | 0.076 | -0.086 | 0.205 | -0.094 | -0.044 | 0.474** | 0.113 | 0.04 | -0.042 | 0.387* |
|  | Pathways in cancer | 0.06 | 0.199 | 0.196 | 0.016 | 0.307 | 0.024 | 0 | -0.056 | 0.227 | -0.075 | -0.068 | 0.474** | 0.096 | 0.05 | 0.007 | 0.445** |
|  | Prion diseases | 0.051 | 0.197 | 0.163 | 0.002 | 0.289 | -0.017 | 0.04 | -0.058 | 0.25 | -0.132 | -0.021 | 0.435** | 0.061 | 0.02 | 0.025 | 0.427** |
|  | Prostate cancer | 0.04 | 0.185 | 0.141 | 0.182 | 0.248 | -0.098 | 0.154 | -0.043 | 0.19 | 0.052 | -0.109 | 0.382* | 0.107 | -0.056 | -0.029 | 0.441** |
|  | Renal cell carcinoma | 0.077 | 0.22 | 0.232 | 0.009 | 0.285 | -0.034 | 0.001 | -0.121 | 0.17 | -0.083 | 0.042 | 0.474** | 0.168 | 0.196 | 0 | 0.366* |
|  | Shigellosis | -0.013 | 0.181 | 0.135 | 0.034 | 0.248 | 0.059 | 0.151 | 0.034 | 0.234 | 0.061 | -0.066 | 0.388* | 0.099 | 0.092 | 0.023 | 0.409* |
|  | Small cell lung cancer | 0.13 | 0.114 | 0.169 | -0.095 | 0.285 | 0.055 | -0.062 | -0.102 | 0.184 | -0.13 | -0.079 | 0.443** | 0.01 | 0.028 | -0.002 | 0.373* |
|  | Staphylococcus aureus infection | -0.043 | 0.154 | 0.048 | -0.064 | 0.295 | -0.096 | 0.306 | -0.068 | -0.05 | -0.078 | 0.245 | 0.285 | -0.124 | -0.187 | -0.005 | 0.164 |
|  | Systemic lupus erythematosus | 0.185 | 0.078 | 0.015 | 0.201 | 0.307 | 0.083 | 0.026 | -0.254 | 0.196 | -0.259 | -0.14 | 0.43** | -0.036 | -0.152 | 0.36* | 0.252 |
|  | Tuberculosis | 0.048 | 0.132 | 0.137 | 0.083 | 0.26 | -0.062 | 0.142 | -0.106 | 0.227 | -0.08 | -0.014 | 0.428** | 0.093 | 0.004 | 0.006 | 0.409* |
|  | Vibrio cholerae infection | -0.274 | -0.11 | 0.222 | 0.115 | -0.148 | -0.223 | 0.159 | 0.087 | -0.003 | 0.162 | 0.178 | -0.084 | 0.178 | 0.075 | 0.013 | -0.106 |
|  | Viral myocarditis | 0.13 | 0.122 | 0.168 | -0.088 | 0.291 | 0.065 | -0.048 | -0.101 | 0.175 | -0.126 | -0.09 | 0.451** | 0.004 | 0.019 | 0.011 | 0.382* |

Spearman analysis was carried out. Values of rho were shown. “*”: *P* < 0.05, “**”: *P* < 0.01. Numbers in red: significant positive correlated with induced OTUs; numbers in green: significant negative correlated with induced OTUs

**Table S4** Sample information

| Sample | Group | CH_2_O(mg.m^-3^) | Time(W) | Container code | Oral input | Elimination |
| --- | --- | --- | --- | --- | --- | --- |
| A.1.1 | A.1 | 0 | 0 | #4 | No | Yes |
| A.1.2 | A.1 | 0 | 0 | #5 | No | No |
| A.1.3 | A.1 | 0 | 0 | #6 | No | No |
| A.1.4 | A.1 | 0 | 0 | #7 | No | No |
| A.1.5 | A.1 | 0 | 0 | #8 | No | No |
| A.2.1 | A.2 | 0 | 0 | #9 | No | No |
| A.2.2 | A.2 | 0 | 0 | #10 | No | Yes |
| A.2.3 | A.2 | 0 | 0 | #11 | No | No |
| A.2.4 | A.2 | 0 | 0 | #12 | No | No |
| A.2.5 | A.2 | 0 | 0 | #13 | No | Yes |
| A.3.1 | A.3 | 0 | 0 | #14 | No | No |
| A.3.2 | A.3 | 0 | 0 | #15 | No | No |
| A.3.3 | A.3 | 0 | 0 | #16 | No | No |
| A.3.4 | A.3 | 0 | 0 | #17 | No | No |
| A.3.5 | A.3 | 0 | 0 | #18 | No | Yes |
| B.1.1 | B.1 | 0.054 | 6 | #4 | Yes | No |
| B.1.2 | B.1 | 0.054 | 6 | #5 | Yes | Yes |
| B.1.3 | B.1 | 0.054 | 6 | #6 | Yes | No |
| B.1.4 | B.1 | 0.054 | 6 | #7 | Yes | No |
| B.1.5 | B.1 | 0.054 | 6 | #8 | Yes | No |
| B.2.1 | B.2 | 0.1 | 6 | #9 | Yes | Yes |
| B.2.2 | B.2 | 0.1 | 6 | #10 | Yes | No |
| B.2.3 | B.2 | 0.1 | 6 | #11 | Yes | No |
| B.2.4 | B.2 | 0.1 | 6 | #12 | Yes | No |
| B.2.5 | B.2 | 0.1 | 6 | #13 | Yes | No |
| B.3.1 | B.3 | 0.25 | 6 | #14 | Yes | No |
| B.3.2 | B.3 | 0.25 | 6 | #15 | Yes | No |
| B.3.3 | B.3 | 0.25 | 6 | #16 | Yes | No |
| B.3.4 | B.3 | 0.25 | 6 | #17 | Yes | No |
| B.3.5 | B.3 | 0.25 | 6 | #18 | Yes | No |
| C.1.1 | C.1 | 0.054 | 12 | #4 | Yes | No |
| C.1.2 | C.1 | 0.054 | 12 | #5 | Yes | Yes |
| C.1.3 | C.1 | 0.054 | 12 | #6 | Yes | Yes |
| C.1.4 | C.1 | 0.054 | 12 | #7 | Yes | No |
| C.1.5 | C.1 | 0.054 | 12 | #8 | Yes | No |
| C.2.1 | C.2 | 0.1 | 12 | #9 | Yes | No |
| C.2.2 | C.2 | 0.1 | 12 | #10 | Yes | No |
| C.2.3 | C.2 | 0.1 | 12 | #11 | Yes | No |
| C.2.4 | C.2 | 0.1 | 12 | #12 | Yes | No |
| C.2.5 | C.2 | 0.1 | 12 | #13 | Yes | No |
| C.3.1 | C.3 | 0.25 | 12 | #14 | Yes | No |
| C.3.2 | C.3 | 0.25 | 12 | #15 | Yes | No |
| C.3.3 | C.3 | 0.25 | 12 | #16 | Yes | No |
| C.3.4 | C.3 | 0.25 | 12 | #17 | Yes | No |
| C.3.5 | C.3 | 0.25 | 12 | #18 | Yes | Yes |
| H1 | H | 0 | 0 | - | Yes | No |
| H2 | H | 0 | 0 | - | Yes | No |
| H3 | H | 0 | 0 | - | Yes | No |
